# Association between an Alzheimer’s Disease-Related Index and *APOE ε4* Gene Dose

**DOI:** 10.1371/journal.pone.0067163

**Published:** 2013-06-26

**Authors:** Frank Schraml, Kewei Chen, Napatkamon Ayutyanont, Roontiva Auttawut, Jessica B. S. Langbaum, Wendy Lee, Xiaofen Liu, Dan Bandy, Stephanie Q. Reeder, Gene E. Alexander, Richard J. Caselli, Adam S. Fleisher, Eric M. Reiman, the Alzheimer’s Disease Neuroimaging Initiative

**Affiliations:** 1 Banner Alzheimer’s Institute and Banner Good Samaritan PET Center, Phoenix, Arizona, United States of America; 2 Department of Mathematics and Statistics, Arizona State University, Tempe, Arizona, United States of America; 3 Department of Psychology, University of Arizona and Evelyn F. McKnight Brain Institute, Tuscon, Arizona, United States of America; 4 Department of Psychiatry, University of Arizona, Tucson, Arizona, United States of America; 5 Department of Neurosciences, University of California San Diego, San Diego, California, United States of America; 6 Department of Neurology, Mayo Clinic Arizona, Scottsdale, Arizona, United States of America; 7 Division of Neurogenomics, Translational Genomics Research Institute, Phoenix, Arizona, United States of America; 8 Arizona Alzheimer’s Consortium, Phoenix, Arizona, United States of America; 9 Departments of Radiology and Psychiatry, Saint Joseph’s Hospital and Medical Center/The Barrow Neurologic Institute, Phoenix, Arizona, United States of America; 10 Department of Radiology, Creighton University School of Medicine, Omaha, Nebraska, United States of America; University of Manchester, United Kingdom

## Abstract

**Background:**

We introduced a hypometabolic convergence index (HCI) to characterize in a single measurement the extent to which a person’s fluorodeoxyglucose positron emission tomogram (FDG PET) corresponds to that in Alzheimer’s disease (AD). *Apolipoprotein E ε4 (APOE ε4)* gene dose is associated with three levels of risk for late-onset AD. We explored the association between gene dose and HCI in cognitively normal *ε4* homozygotes, heterozygotes, and non-carriers.

**Methods:**

An algorithm was used to characterize and compare AD-related HCIs in cognitively normal individuals, including 36 *ε4* homozygotes, 46 heterozygotes, and 78 non-carriers.

**Results:**

These three groups differed significantly in their HCIs (ANOVA, *p* = 0.004), and there was a significant association between HCIs and gene dose (linear trend, *p* = 0.001).

**Conclusions:**

The HCI is associated with three levels of genetic risk for late-onset AD. This supports the possibility of using a single FDG PET measurement to help in the preclinical detection and tracking of AD.

## Introduction

Fluorodeoxyglucose (FDG) positron emission tomography (PET) measurements of the cerebral metabolic rate for glucose (CMRgl) provide a neuroimaging biomarker for the detection and tracking of Alzheimer’s disease (AD) [1] and for distinguishing Alzheimer’s disease (AD) from other dementias (e.g. frontotemporal dementia and dementia with Lewy bodies). This biomarker is also efficacious in predicting future AD in persons with mild cognitive impairment (MCI), as well as cognitively normal individuals [2]. The potential value of preclinical identification and tracking of AD is receiving increasing interest with the goal of preventative intervention [3], and it is this which has sparked recent research endeavors.

In that the apolipoprotein E (*APOE*) *ε4* allele is the major genetic risk factor for late-onset Alzheimer’s disease (AD) [4] and the *APOE ε4* gene dose (each additional copy of the *ε4* allele in a person’s APOE genotype) is associated with a greater risk of AD and a younger age at clinical onset [5], we previously used FDG PET in 160 cognitively normal late-middle-aged *APOE ε4* homozygotes, heterozygotes, and noncarriers to characterize associations between *APOE ε4* gene dose and lower CMRgl in precuneus, posterior cingulate, parietal, temporal, and frontal regions that are preferentially affected in patients with AD [6].

In this previous study, we were able to demonstrate that *APOE ε4* gene dose was significantly correlated with hypometabolism in postulated brain regions. Our methodology entailed use of preselected regions of interest (ROI’s) with its inherent limitation of inflated Type I error due to multiple regional comparisons. We recently developed a voxel-based data analysis method that uses all of the data in a person's FDG positron emission brain tomogram to produce a single measurement, an “AD-related hypometabolic convergence index (HCI)” [7]. This HCI characterizes the extent to which the pattern and magnitude of a person’s brain FDG alterations, relative to a normal control (NC) group, correspond to the pattern and magnitude of the brain alterations in AD patients. Because this methodology capitalizes on as much of the data in the image as possible (rather than one or more preselected ROIs), it overcomes the problem of inflated Type I error due to multiple regional comparisons.

In this study, we used HCI’s for the same cognitively normal late-middle-aged cohort with varying doses of *APOE ε4*, along with reference data from the AD Neuroimaging Initiative (ADNI) to characterize these individuals, with the aim of determining if this single FDG PET measurement could be used to characterize an association between three levels of genetic risk of AD prior to the onset of symptoms. Here we report that the HCI is associated with three levels of genetic risk for late-onset AD.

## Materials and Methods

### Ethics Statement

All participants provided written informed consent, and agreed not to be given any information about their *APOE* genotype. All parts of the study were conducted in the United States with IRB approval from The Banner Good Samaritan Hospital Institutional Review Board Committee (Affiliated with: Banner Health).

### Participants

This study capitalized on data originally acquired in 160 cognitively normal persons 47–68 years of age, who had a reported first degree family history of Alzheimer’s dementia, and whose FDG PET images were originally analyzed using a voxel-based brain mapping algorithm, as described in a previous report [6]. Venous blood samples were drawn, and *APOE* genotypes were characterized with analysis by restriction fragment-length polymorphisms [8]. This cohort included 36 *APOE ε4* gene homozygotes (HM), 46 *ε4* heterozygotes (HT) (all with *ε3*/*ε4* genotype), and 78 *ε4* noncarriers (NC) (60 with *ε3*/*ε3* genotype and 18 with *ε2*/*ε3* genotype). The subjects denied any memory or other cognitive skill impairment, and had scores of at least 28 on the Folstein Mini Mental State Examination [9] and <10 on the Hamilton Depression Rating Scale (HAM-D) [10]. They did not satisfy criteria for a current psychiatric disorder using a structured psychiatric interview [11], and had a normal neurological examination. The subjects were fully evaluated with clinical ratings and a battery of neuropsychological tests, as well as volumetric magnetic resonance imaging (MRI) and FDG brain PET; and none of the participants had used psychoactive medications for at least 2 weeks before their PET scan.

### FDG PET

FDG PET was performed with 951/31 ECAT scanner (Siemens, Knoxville, Tennessee) as previously described [6]. The acquisition protocol included a 20-minute transmission scan, the intravenous injection of 10 millicuries (mCi) (370 megabecquerels) of 18F-fluorodeoxyglucose and a 60-minute dynamic sequence of emission scans. The subjects had fasted for at least 4 hours prior to the study, and lay quietly in a darkened room with eyes closed and directed forward. FDG PET images were reconstructed using the 30–60 minute emission scans, filtered back projection, a Hanning filter of 0.40 cycles per pixel and measured attenuation-correction, resulting in 31 slices with in-plane resolution of about 8.5 mm full width at half maximum (FWHM), an axial resolution of 5.0–7.1 mm FWHM, a 3.375 mm slice thickness and a 10.4 cm axial field of view. SPM5 (http://www.fil.ion.ucl.ac.uk/spm/) was used to deform each individual’s FDG PET image into the coordinate space of the Montreal Neurological Institute (MNI) template and smoothed using a Gaussian filter with 15 mm FWHM.

### Alzheimer’s Disease Neuroimaging Initiative (ADNI) Dataset

Data from a separate group of individuals participating in the Alzheimer’s Disease Neuroimaging Initiative (ADNI) [12] study, a longitudinal multisite study supported by the National Institutes of Health, private pharmaceutical companies, and nonprofit organizations, with approximately 50 medical center and university sites across the United States and Canada (www.loni.ucla.edu/ADNI), was used as an independent data set to establish a template for defining patterns of regional hypometabolism associated with AD for the HCI computations. Their baseline FDG PET scans were downloaded from the ADNI Laboratory on Neuroimaging (LONI) website (www.loni.ucla.edu/ADNI). This group included 68 subjects with probable AD and 78 elderly healthy controls (HL). Individuals in the AD group had Mini-Mental State Examination (MMSE) [9] scores of 20–26, had a Clinical Dementia Rating (CDR) global score [13] of 0.5 or 1.0, and met NINCDS/ADRDA criteria for probable AD [14]. HL had MMSE scores of at least 24, a CDR global score of 0; and no diagnosis of depression, Mild Cognitive Impairment (MCI), or dementia. Additional inclusion and exclusion criteria, including lists of approved medications and cognitive test measures for inclusion, can be found on the ADNI website (www.loni.ucla.edu/ADNI).

Each participating ADNI site acquired and reconstructed FDG-PET data with the use of measured-attenuation correction and the specified reconstruction algorithm for each scanner type according to a standardized protocol (www.loni.ucla.edu/ADNI/Data/ADNI_Data.shtml). All images were pre-processed by ADNI PET Coordinating Center investigators at the University of Michigan and uploaded to the LONI ADNI website. These images were downloaded by investigators at Banner Alzheimer’s Institute for additional pre-processing using SPM5 (http://www.fil.ion.ucl.ac.uk/spm).

### Computation of Hypometabolic Convergence Index (HCI)

FDG brain PET images from the 68 ADNI subjects with probable AD were compared to those of 78 ADNI healthy controls using a voxel-wise two-sample independent t-test implemented in SPM5. In doing so, the image from each subject was normalized for individual variations in whole brain measurements using proportionate scaling. The resulting AD hypometabolic map (the t-map of lower CMRgl in AD patients) was then transformed into a z-score map of AD-like hypometabolism.

In addition to this AD hypometabolic map, the FDG-PET images from 101 ADNI healthy controls (2 HMs, 24 HTs and 75 NCs of APOE ε4) served as an independent normative database, and were used to characterize the extent of regional CMRgl reductions in each cognitively normal person with variable genetic risk for LOAD in our Arizona cohort. Again, a two-sample t-test was conducted comparing the image of a single individual in our Arizona cohort to ones of the normative database (the single subject his/herself as one group). Similarly, the T-score map was generated for this subject and converted also to a z-score map as previously described [7]. Thus, each member of our separate cohort of cognitively normal individuals with varying doses of the *APOE ε4* gene had his or her FDG PET comparative hypometabolic map**.** This hypometabolic map and the above-noted AD-like hypometabolic map were then used to calculate an HCI for this subject; essentially as the sum of the product of z-scores from each cerebral voxel [7].

### Statistical Analysis

The three genetically distinct *APOE ε4* gene groups-HM, HT and NC were compared in terms of their age, gender, educational level, and performance on each of the neuropsychological measures. A comparison of the HCI scores, using analysis of variance (ANOVA), was performed, and Z-score voxel-wise comparisons of AD-associated regions of hypometabolism were produced for each diagnostic group. Two-sample t-tests (assuming equal variances) were also performed on the HCI scores between the groups as follows: NC and HT, NC and HM, and HM and HT. Regression analysis was performed demonstrating the association between HCI z-scores and *APOE ε4* gene dose, and Pearson correlations were used to evaluate the relationship between HCI index scores and cognitive measures.

## Results

**Figure 1:**
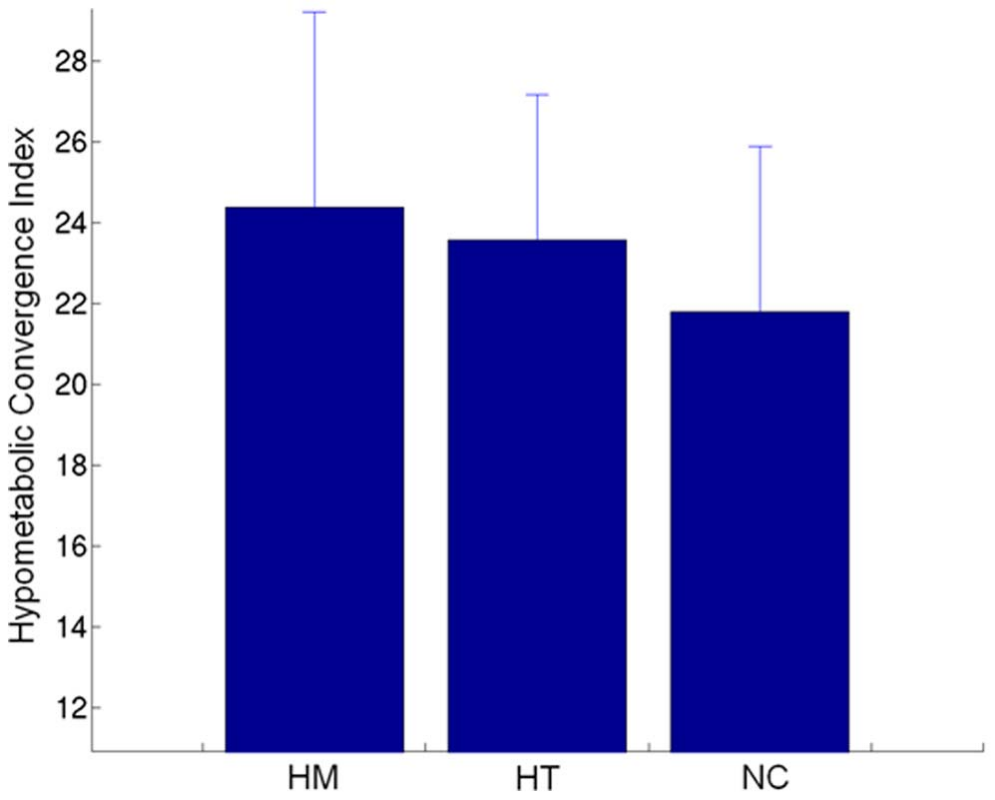
Mean Hypometabolic Convergence Index (HCI) for APOE ε4 homozygotes (HM), heterozygotes (HT) and non-carriers (NC) showed a positive association between the HCI and the *APOE ε4* gene dose.

**Figure 2:**
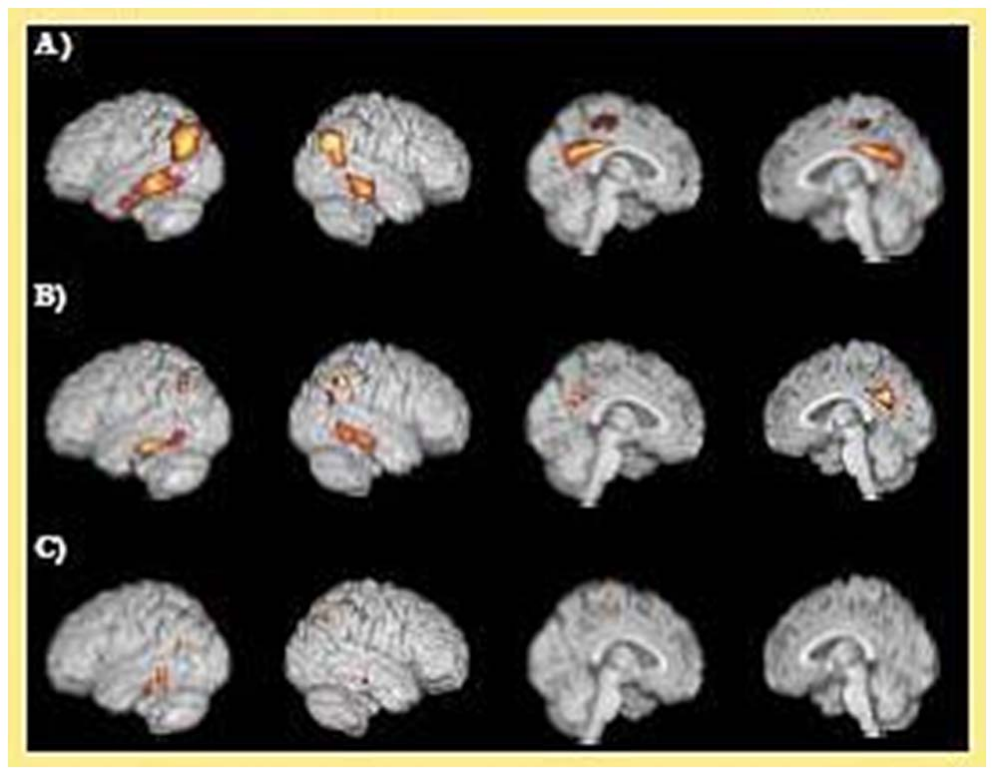
HCI z-score maps of AD-associated hypometabolism in a APOE ε4 homozygote (A), a APOE ε4 heterozygote (B) and a APOE ε4 non-carrier (C) demonstrate differences in extent of hypometabolism proportional to APOE ε4 gene dose.

As shown in Table 1, The HM, HT, and NC groups did not differ significantly in their age, sex, or educational level. There were, likewise, no significant differences in neuropsychological test performance with the exception of long term memory on the AVLT [15], with trends in short term memory (all within age/education adjusted normal limits). Significant differences were seen in the group’s HCI’s (24.38±4.84, 23.58±3.59, and 21.80±4.09, respectively for HM, HT and NC; ANOVA, *p* = 0.004), as shown in Figure 1. Additionally, there was a significant association between HCI’s and the *APOE ε4* gene dose (linear trend, *p* = 0.001). Two sample t-tests performed on the HCI scores between NC and HT, NC and HM, and HM and HT, respectively, revealed significant differences between the HCI scores of the NC and HT groups (p = 0.013) and the NC and HM groups (p = 0.004), while no difference was found between the HM and HT groups. The product image of the AD group hypometabolism map (the z-score map) with the one from an individual is helpful visually to see how these two are similar. Figure 2 shows such product maps in a representative HM (row A), HT (row B) and NC (row C) subject. There were no significant associations between HCI scores and any cognitive measures.

**Table 1:** Subject characteristics, clinical ratings, and neuropsychological test scores.

Characteristic, rating, or test score	*ε*4Homozygotes	*ε*4Heterozygotes	*ε*4noncarriers	*P* value
	*n* = 36	*n* = 46	*n* = 78	
Age, years	55.6±5.2	56.1±4.2	56.5	0.52
Gender (F/M)	23/13	30/16	49/29	0.96
Years of Education	15.8±2.5	16.0±2.6	15.8±2.3	0.99
MMSE score	29.7±0.6	29.9±0.4	29.6±0.7	0.38
HAM-D	2.5±3.3	1.7±1.9	3.9±4.7	0.08
Auditory verbal learning test scores				
Total learning	47.6±8.0	48.5±9.4	50.2±10.2	0.30
Short-term recall	9.4±2.5	10.4±2.5	10.3±3.4	0.07
Long-term recall	8.7±2.9	10.0±2.9	9.8±3.4	0.05
Complex figure test scores				
Copy	34.9±1.5	33.9±2.9	34.3±1.9	0.10
Recall	18.4±6.1	19.0±6.6	17.6±6.1	0.60
Boston naming test	57.1±3.1	56.8±2.9	56.7±3.4	0.67
Wechsler Adult Intelligence Scale-Revised scores				
Information	11.9±2.2	12.3±2.1	11.2±2.1	0.06
Digit span	11.3±2.2	11.6±2.9	11.3±2.7	0.81
Block design	11.9±2.7	12.2±2.7	11.9±2.5	0.83
Mental arithmetic	11.9±2.4	12.3±2.5	11.4±2.7	0.47
Similarities	12.4±2.2	12.5±2.0	11.8±1.9	0.28
Controlled oral word association test	44.4±11.0	43.8±11.5	47.7±10.3	0.23
Wechsler memory Scale-Revised orientation subtest score	13.8±0.4	13.9±0.4	13.7±0.4	0.51

Values are means ± SD with ranges in parentheses as appropriate. *P* values were calculated using the unpaired 2-tailed *t*-tests or 2-tailed Pearson χ^2^ test, uncorrected for multiple comparisons.

## Discussion

This study demonstrates an association between AD-related HCIs and *APOE ε4* gene dose in a large group of previously studied, cognitively normal, late-middle-aged *ε4 homozygotes, heterozygotes,* and *non-carriers* from the Arizona *APOE* cohort, and indeed, significant differences among the three genetic groups. It illustrates the possibility of characterizing the AD-related pattern of cerebral hypometabolism in the preclinical detection of AD using a summary index that is free from the Type I error associated with multiple regional comparisons.

Whereas our previous publication focused on the HCI in the context of distinguishing AD, MCI and normal controls, and predicting MCI to AD conversion [7], our current study served as a performance test of the HCI in cognitively normal individuals and investigated its potential to distinguish these individuals with different levels of genetic risk of AD. With the recently proposed research criteria for preclinical AD, based on biomarker measurements, there will be increasing interest in ways to characterize AD biomarkers in a single sensitive measurement. To the extent to which they are able to characterize the preclinical stages of AD, these strategies may have potentially important roles in research and clinical settings. Additional studies are needed to determine the extent to which the HCI, alone or in combination with other genetic or biomarker data, can help predict a person’s subsequent clinical course, predict a differential response to treatment, help to enrich prevention trials with those at highest clinical risk, predict a differential response to future preclinical AD treatments, or help in the evaluation of genetic and non-genetic risk factors for AD.

We recognize a number of limitations to our study: These include a significant overlap between the three group’s HCI’s, suggesting relatively low sensitivity for the preclinical detection of AD and the potential for refinement of this summary index measurement. More information is also needed to determine the extent to which the HCI, alone or in combination with other genetic or biomarker measurements, is predictive of clinical course or response to treatment; and, therefore, this summary index measurement should not be used to predict an individual’s risk for development of AD. Moreover, it is difficult based on the evidence from FDG-PET alone to support, for example, a specific pathological abnormality of preclinical AD without other data showing Aβ-amyloid pathology. In general, FDG-PET-revealed-abnormalities could be related to a reduction in the activity or density of the terminal neuronal fields or perisynaptic glial cells that innervate these regions, a metabolic dysfunction, or a combination of these factors [16]. We also note that the HCI results from this study may not be directly compared to those in our previous study on MCI and AD subjects as the normal database used in this study is larger than that of the previous study. However, to evaluate the reliability of the HCI measurement, we have examined the differences in the HCI results using these two overlapping, but different normal databases, and found that all statistical significances observed are identical using either normal database. Finally, our group has not yet applied this summary index measurement for the tracking of longitudinal change in our healthy subjects and in patients with AD or MCI. We are working to extend this index to be applicable to longitudinal data and will report the findings in future separate studies.

It is also important to acknowledge alternative approaches to the characterization of AD in a single ROI or voxel-based index. For example, Minoshima et al. [17] demonstrated that a ROI encompassing the posterior cingulate cortex consistently indicated significant FDG uptake (glucose metabolic) reduction in patients with very early Alzheimer’s disease, and demonstrated utility regarding prediction and analysis of actual patients.

Landau et al. [18] developed a single “metaROI” reflective of glucose hypometabolism in specific brain regions established as hypometabolic in previous FDG PET studies of AD and MCI, and showed that radiotracer uptake in this composite ROI was a sensitive measure of change in cognition and functional ability in AD and MCI, and had value in predicting future cognitive decline [19]. Herholz et al. [20], also using FDG PET data, derived an AD summary index measure referred to as the “AD t-sum”, indicative of the severity of the hypometabolism in those brain areas typically affected by AD, and demonstrated its utility in monitoring the progression of MCI to AD [21]. In a recent comparison study [22], HCI, metaROI and AD t-sum based PALZ techniques performed comparably in differentiating AD, MCI and healthy controls. Future studies are needed to compare these indices and others in terms of the sensitivity of differentiating cognitively normal individuals with different levels of AD risk.

The potential roles of brain imaging techniques depend not only on the imaging modality used, but the way that the data are analyzed. We anticipate that summary brain imaging measurements like the HCI will continue to be developed, tested, and applied in the preclinical study of AD.
